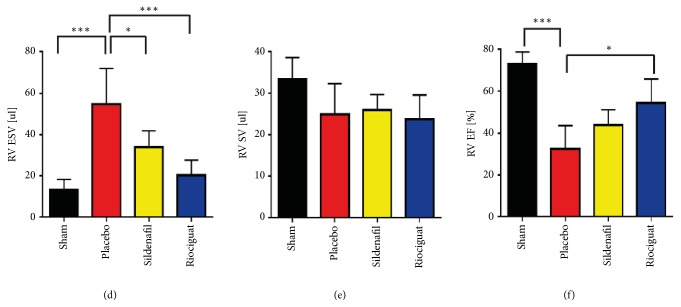# Response to: Comment on “Effect of Riociguat and Sildenafil on Right Heart Remodeling and Function in Pressure Overload Induced Model of Pulmonary Arterial Banding”

**DOI:** 10.1155/2018/7491284

**Published:** 2018-12-16

**Authors:** Nabham Rai, Swathi Veeroju, Yves Schymura, Wiebke Janssen, Astrid Wietelmann, Baktybek Kojonazarov, Norbert Weissmann, Johannes-Peter Stasch, Hossein Ardeschir Ghofrani, Werner Seeger, Ralph Theo Schermuly, Tatyana Novoyatleva

**Affiliations:** ^1^Universities of Giessen and Marburg Lung Centre (UGMLC), Aulweg 130, 35392 Giessen, Germany; ^2^German Center for Lung Research (DZL), 35392 Giessen, Germany; ^3^Max-Planck-Institute for Heart and Lung Research, Ludwigstrasse 43, 61231 Bad Nauheim, Germany; ^4^Bayer Pharma AG, Aprather Weg 18a, 42096 Wuppertal, Germany; ^5^Institute of Pharmacy, Martin Luther University of Halle-Wittenberg, Wolfgang-Langenbeck- Strasse 4, 06120 Halle (Saale), Germany

We would like to thank Dr. Andersen for careful reading and the comment [1] on the manuscript “Effect of Riociguat and Sildenafil on Right Heart Remodeling and Function in Pressure Overload Induced Model of Pulmonary Arterial Banding” by Rai et al. [2]. As described in our manuscript, data like stroke volume (SV) or ejection fraction (EF) are calculated from the individual end diastolic and systolic volumes (paired analysis). However, we recalculated the stroke volume and the ejection fraction and identified an error in values for the end systolic volumes (ESV) of the animals treated with riociguat, which explains the difference in the stroke volume that has been found. We identified that two numbers were mistakenly entered in the calculation of the mean ESV value of the riociguat group. This resulted in the mean ESV value of 24.7 ± 10.1*μ*l which was given in the manuscript. The corrected mean ESV value for riociguat is 20.3 ± 7.4*μ*l (if these 2 numbers are not included) (Figure 1(d)). Further, the calculation of SV and EF included mistakes in the excel file that has been used for the generation of the graphs and the statistics in Graphpad Prism. Based on the given EDV and ESV values, we recalculated the SV and the EF and found some changes which we would like to correct. The SV of the riociguat group changed to 23.7 ± 5.8% (reported value 28.5 ± 7.3%) and of the EF to 54.3% ± 11.4% (reported value 57.6 ± 8.6%). The SV of the placebo group changed to 24.9 ± 7.4*μ*l (reported value: 24.2 ± 7.3*μ*l) and the EF to 32.4 ± 11.1% (reported value: 30.0 ± 9.5%). The SV of the sildenafil group changed to 26.0 ± 3.7*μ*l (reported value: 25.9 ± 3.6*μ*l) and the EF to 43.8 ± 7.4% (reported value: 44.9 ± 4.9%). There were no changes in the Sham group (Figures 1(e) and 1(f)). There is no change in the interpretation and the conclusions drawn from the results [2].

Another point that has been raised by Dr. Andersen is an increased mortality that he has observed in a similar experimental design in rats with pulmonary artery banding treated with sildenafil and/or Bay41-2272, an early precursor molecule of riociguat [3]. In our initial report on the effects of Bay41-2272 on experimental pulmonary hypertension in monocrotaline-injected rats, we did not observe a significant effect on mortality (rather an improvement) at a dose of 10 mg/kg per day [4]. In our recent study, we have also not observed any impact of sildenafil or riociguat on mortality of the animals. However, the used species (rat versus mouse) or strain, the degree of pulmonary arterial stenosis, or the way of administration of the compounds can influence the outcome of a study. In general, sildenafil and riociguat are well tolerated in patients with pulmonary vascular disease; however, the combination of both compounds displayed some unfavorable safety signals in patients with pulmonary arterial hypertension [5].

## Figures and Tables

**Figure 1:**